# Digitized Physical Impressions Optimize Apical Adaptation of Bio-Root Inlays for Apexification: A Comparison of Four Impression Techniques

**DOI:** 10.3390/dj14090568

**Published:** 2026-09-04

**Authors:** Yasser Alsayed Tolibah, Wahid Juha, Mhd Kheir Awad, Mohammad Tamer Abbara, Marwan Alhaji, Jihad Abou Nassar, Osama Aljabban, Nada Bshara, Ziad D. Baghdadi

**Affiliations:** 1 Department of Pediatric Dentistry, Damascus University, Damascus P.O. Box 3062, Syriamrwan.2013.55.a@gmail.com (M.A.); gmmn2012@gmail.com (N.B.); 2 Department of Endodontics, Aleppo University, Aleppo P.O. Box 3062, Syria; juhawaheed1995@gmail.com; 3 Department of Restorative Dentistry, Faculty of Dentistry, Damascus University, Damascus P.O. Box 3062, Syria; mhmawad241995@gmail.com; 4 Department of Endodontics, University of Kalamoon, Damascus P.O. Box 3062, Syria; tamerabbara@gmail.com; 5 Department of Fixed Prosthodontics, Damascus University, Damascus P.O. Box 3062, Syria; drjnassar@gmail.com; 6 Department of Endodontics, Damascus University, Damascus P.O. Box 3062, Syria; dr.ossamaljabban@gmail.com; 7 Top Smiles Pediatric Dentistry & Orthodontics, 246 St. Anne’s Road, Winnipeg, MB R2M 3A4, Canada; 8 Butterfly Dental Group, Winnipeg, MB R3Y 1P5, Canada; 9 Children’s Dental Centre, 1630 Ness Ave., Winnipeg, MB R3J 3X1, Canada

**Keywords:** bio-root inlay, cone-beam computed tomography, digital workflow, immature teeth, intraoral scanner

## Abstract

**Objectives**: To evaluate the three-dimensional adaptation of prefabricated Bio-Root inlays fabricated from impressions of simulated immature permanent root canals using four techniques: direct intraoral scanning (A), a light-body rubber impression digitized with an intraoral scanner (B), cone-beam computed tomography (CBCT)-based design (C), and a conventional indirect technique (D). **Methods**: Ten standardized simulated immature roots (extracted mandibular premolars prepared to a 1.5 mm apical diameter and 10 mm length) were used in a within-specimen comparative design, with each root receiving inlays fabricated by all four techniques. After fabrication and seating of the bioceramic inlays, the void percentage around each inlay was quantified by CBCT at the coronal, middle, and apical thirds relative to the baseline empty-canal volume. Data were analyzed using the Friedman, Wilcoxon signed-rank (Bonferroni-corrected), and linear mixed-model tests (α = 0.05). **Results**: Technique significantly affected void percentage (F(3,98) = 12.87, *p* < 0.001). Pooled across all canal thirds, Technique B had the lowest median void percentage (6.00 [7.00]), followed by A (8.00 [8.75]), C (12.00 [12.50]), and D (12.00 [19.00]). Independently of technique, the median void percentage increased from the coronal (6.00) to the middle (11.00) and apical (19.00) thirds. Differences among techniques were non-significant coronally (*p* = 0.437) but significant in the middle (*p* = 0.014) and apical (*p* < 0.001) thirds, with Technique B providing the best apical adaptation. **Conclusions**: The impression method significantly affected inlay adaptation. A light-body rubber impression digitized with an intraoral scanner yielded the best overall and apical fit, supporting digital workflows, particularly those using digitized physical impressions, for apexification. Adaptation deteriorated toward the apex regardless of technique.

## 1. Introduction

Immature necrotic permanent teeth pose a unique challenge for dental practitioners because of their complex treatment requirements. Various materials have been introduced to enhance apical closure, including calcium silicate-based cements. Modern materials in this family, such as bioceramics, offer improved handling properties and are commercially available in pre-mixed forms, facilitating their use in endodontic procedures and reducing discoloration compared with earlier generations [1,2,3].

The delivery and condensation systems used for calcium silicate-based cements significantly affect the sealing ability of the root canal system, a critical determinant of endodontic success [1,4]. Bio-Root inlays represent a novel approach to apexification that does not require conventional delivery and condensation systems: an impression of the immature root canal space is taken and used to fabricate a custom mold filled with calcium silicate cement. After setting, the material forms a ready-to-use inlay that conforms precisely to the root canal space, streamlining the conventional steps of material delivery and condensation with calibrated pluggers [5,6].

This concept has been further developed to incorporate a fiber post into the Bio-Root inlay. The post facilitates handling and placement within the canal, ensuring a precise fit before sealing the interface with an appropriate sealer. It also supports subsequent coronal restoration, particularly for extensively damaged immature teeth [7].

To date, despite the technical advancement of the Bio-Root inlay concept, no preferred method has been established for obtaining impressions of immature permanent root canals, despite the various techniques described in the literature.

It has been reported that impressions of immature permanent root canals can be obtained using light silicone [5,6,7]. Digital scanners have also been proposed for recording impressions when designing cores and post-and-cores for prepared canals [8,9]. Furthermore, the canal space shape can be captured using three-dimensional (3D) radiographic images obtained through cone-beam computed tomography (CBCT). This has been used in endodontic treatments to determine the working length accurately [10], evaluate canal shapes before and after preparation, assess the efficiency of preparation files [11], guide drilling of calcified canals [12], create 3D-printed macro-models of teeth for educational purposes [13], and detect obturation gaps and voids [14].

This article introduces a novel research concept not previously reported in the literature, aiming to advance apexification treatment by integrating digital workflows. The proposed methodology focuses on optimizing the acquisition of immature root canal morphology for fabricating Bio-Root inlays. Three digital approaches are proposed: designing the inlay from a direct intraoral scan of the immature root canal, from cone-beam computed tomography (CBCT) data, or from a digitally scanned light-body silicone impression. These digital techniques will be evaluated alongside a conventional impression-based method, which serves as the control.

While a perfect void-free interface is theoretically ideal, in clinical practice the residual void space is typically filled with the bioceramic sealer. However, minimizing sealer thickness and interfacial voids remains important because thicker sealer films are generally more susceptible to dissolution and microleakage, potentially compromising the long-term seal [1,3,14].

The null hypothesis was that all Bio-Root inlay design methods would produce inlays with comparable three-dimensional adaptation within the canal.

## 2. Materials and Methods

### 2.1. Ethical Statement and Settings

This in vitro within-specimen comparative study received ethical approval from Damascus University’s Local Research Ethics Committee (Approval No. UDDS-361-13032023/SRC-2654) and was reported in accordance with the Preferred Reporting Items for Laboratory Studies in Endodontology (PRILE) 2021 guidelines. It was funded by Damascus University (funder No. 501100020595) and conducted in accordance with the Declaration of Helsinki.

### 2.2. Sample-Size Calculation

Because this model had not been investigated previously, the sample size was determined a priori using a Monte Carlo (simulation-based) power analysis (R package simr v1.0.7, built on lme4). The simulation specified technique as a fixed effect (four levels) and specimen as a random intercept to capture the within-specimen correlation of repeated measurements; in the absence of comparable prior data, a large technique effect and a moderate between-specimen variance were assumed. Across 1000 simulations, a sample of 10 specimens, each contributing 12 within-specimen measurements (120 observations in total [10 teeth × 4 techniques × 3 canal levels]), yielded a power of 91.4% (95% CI, 89.5–93.1%) to detect the technique effect at α = 0.05. The large technique effect subsequently observed in the linear mixed model (F(3,98) = 12.87, *p* < 0.001) was consistent with this assumption and confirmed that the study was adequately powered.

### 2.3. Sample Selection and Preparation

Ten intact, mature, permanent human mandibular premolars, recently extracted for orthodontic reasons, were included; patients had provided informed consent for the use of their extracted teeth. Each premolar was examined under a 2.5× magnification lens (Carson handheld; Ronkonkoma, New York, NY, USA) to confirm the absence of cracks, and a periapical radiograph confirmed the absence of anatomical abnormalities.

The crown was trimmed to a level 2 mm above the cemento-enamel junction, and the apical root was sectioned so that each sample was 10 mm long. The canal was explored with a size-10 K-file and then prepared with rotary files (ORODEKA Ltd., Xincheng, Jining, China) up to 20/.06. It was subsequently enlarged with Peeso reamers up to size 5, standardizing all apices to a 1.5 mm diameter to simulate an immature open apex. The canal was irrigated with 3 mL of 5.25% sodium hypochlorite (NaOCl; Merck, Darmstadt, Germany) between each file and each Peeso reamer (Erskine Dental, Macksville, New South Wales, Australia). Final irrigation comprised 10 mL of 5.25% NaOCl, 5 mL of saline, and 2 mL of 17% EDTA (Dentsply Tulsa Dental, Tulsa, OK, USA). The canals were then rinsed with 5 mL of saline, 5 mL of 5.25% NaOCl, and 5 mL of saline.

**Figure 1:**
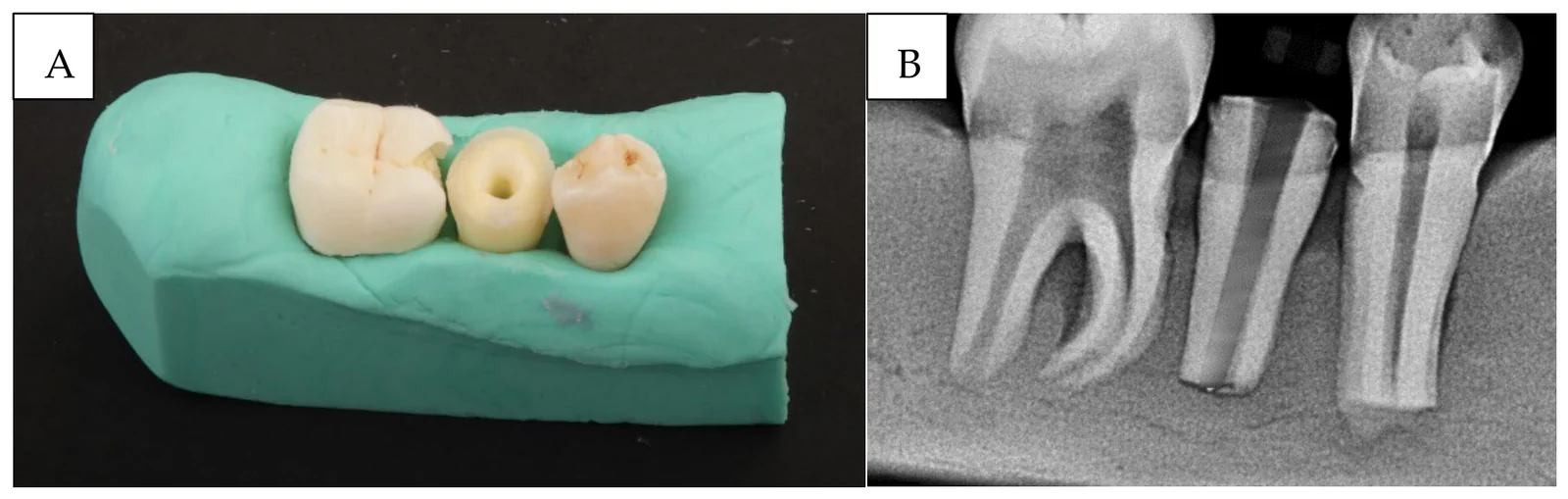
The specimen embedded in heavy-body putty rubber and flanked by two adjacent teeth to simulate the intraoral anatomical situation. (**A**) Photographic view; (**B**) Periapical radiograph.

Each premolar was embedded in a heavy-body rubber putty block with two adjacent teeth (a premolar and a mandibular molar) to simulate the intraoral anatomical conditions during impression-taking and scanning (Figure 1).

### 2.4. Impression Techniques and Inlay Fabrication

Using a within-specimen comparative design, each of the ten standardized roots was subjected to all four techniques (A–D), so that each root served as its own control.

**Figure 2:**
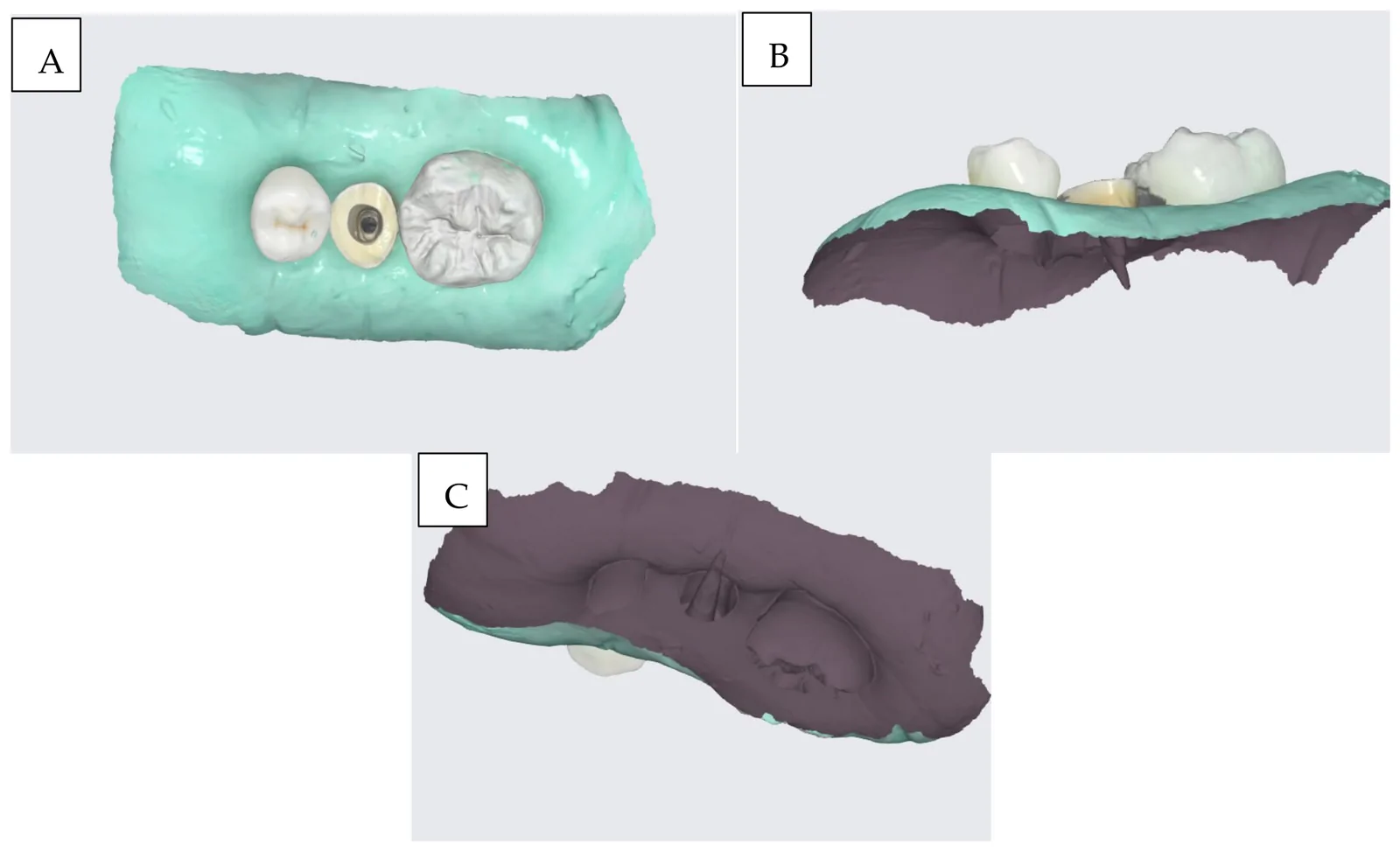
Three-dimensional surface obtained with the intraoral scanner from the immature canal impression. (**A**) Occlusal (top) view; (**B**) Lateral (side) view; (**C**) Apical (bottom) view.

**Technique A—Direct intraoral scanning (IOS).** The immature canal space was scanned directly with an intraoral scanner (Medit i700; Medit Corp., Seoul, Republic of Korea) using the maximum 23 mm depth-of-field setting and the dedicated “model digitizing” filter. Scanning proceeded from the coronal orifice toward the apex until the entire canal wall was captured, and the surface was exported as an STL file (Figure 2).

**Figure 3:**
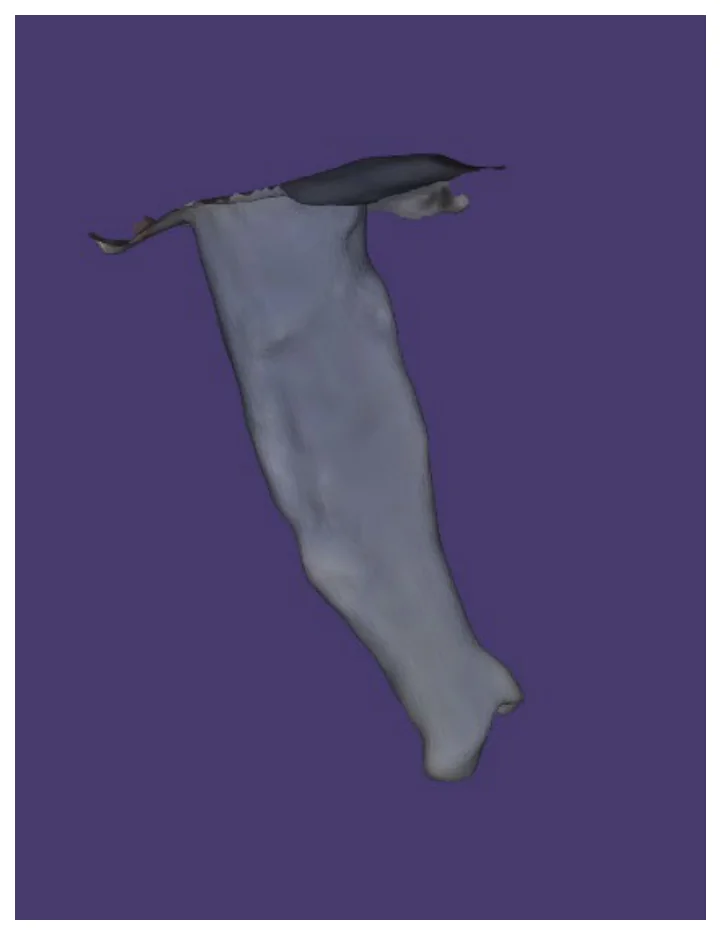
Three-dimensional model of the light-body silicone impression of the root canal, obtained after digital scanning.

**Technique B—Digitized soft-rubber impression.** A light-body rubber impression material (Zetaplus system; Zhermack, Badia Polesine, Italy) was placed in the canal, and a size-80 K-file was advanced to the working length to carry and reinforce the impression. After the material fully set, the impression was removed and digitized with the same intraoral scanner (Medit i700), which captured the fine detail of the canal replica (Figure 3). The scan was then exported as an STL file.

**Figure 4:**
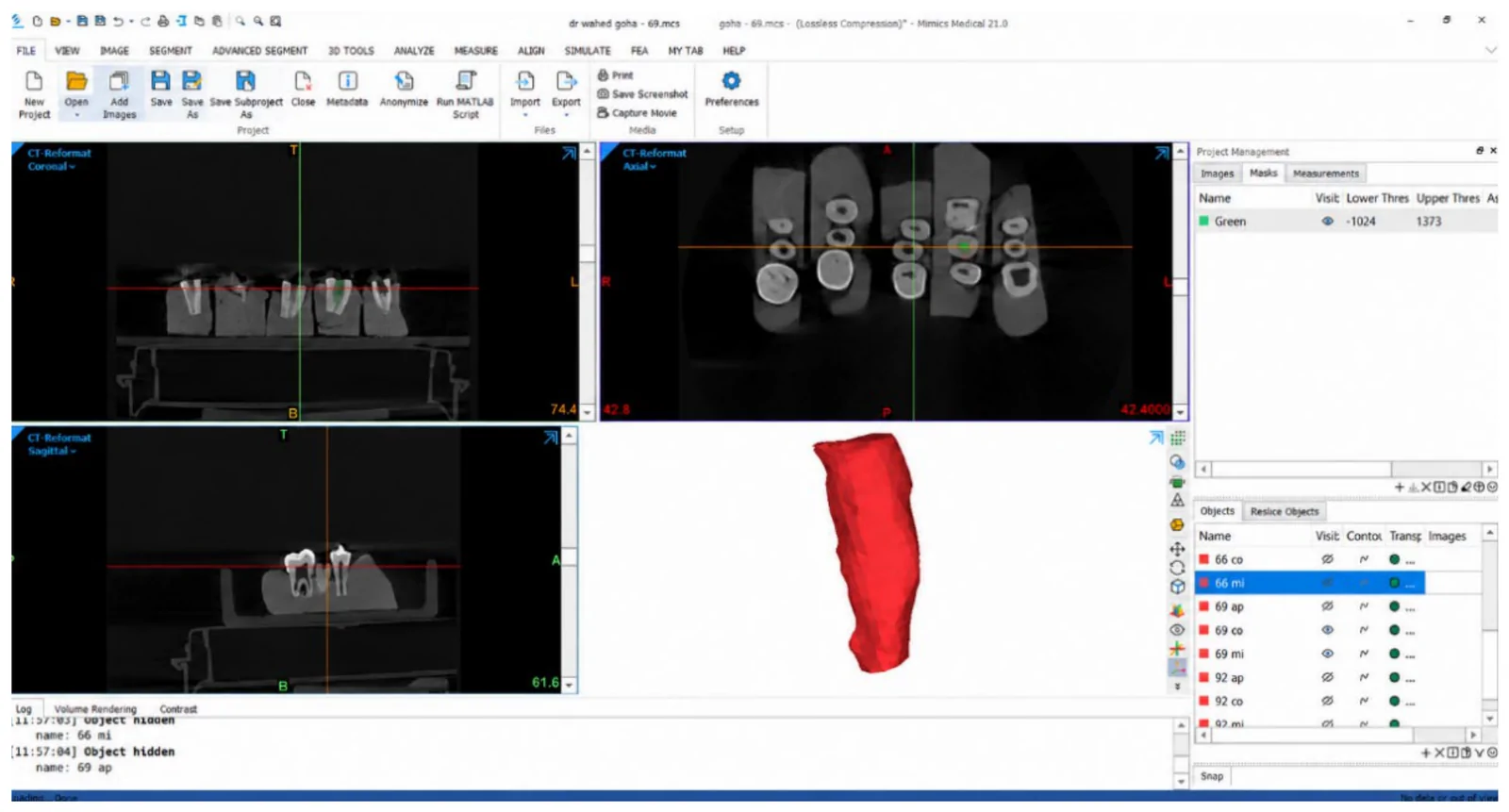
Three-dimensional model of the root canal after CBCT segmentation using Mimics Software.

**Technique C—CBCT-based design.** The canals were imaged with cone-beam computed tomography (PHT-6500; Vatech, Hwaseong-si, Republic of Korea) using a 120 × 90 mm field of view, 120 kV, 15 mA, a 0.2 mm voxel size (standard-resolution mode), and a 24 s exposure time. The cavity boundaries of the simulated immature canal were segmented on the Hounsfield-unit scale in Mimics Research (v21.0.0.406; Materialise NV, Leuven, Belgium). The segmented cavity was then converted into a lock-and-key mold using the same digital procedure described above (Figure 4).

**Figure 5:**
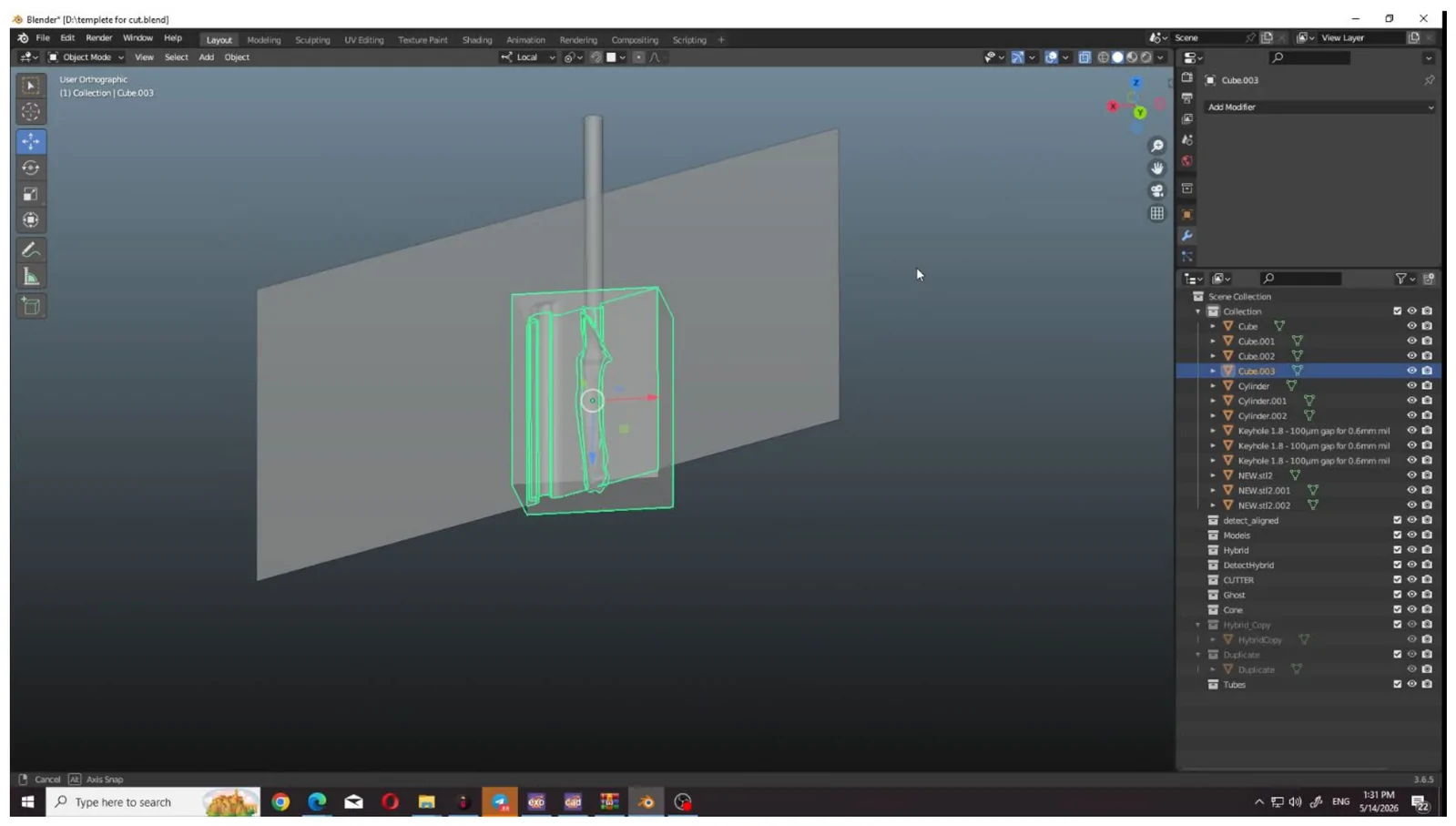
Conversion of the digital impression into an inverse mold model in Blender.

**Common digital design workflow (Techniques A, B, and C).** STL data were imported into Blender (v4.2; Blender Foundation, Amsterdam, The Netherlands), and the region of interest was delineated from the cemento-enamel junction to the apex. The canal replica was converted into an inverse (negative) model using the Boolean Difference tool. The Smooth Brush and Voxel Remesh tools were used to eliminate internal undercuts and protrusions and to generate a smooth cavity that allows unobstructed insertion of the prefabricated Bio-Root inlay. A lock-and-key mold was then designed to split along a plane parallel to the inlay’s long axis. The Boolean Difference tool was used to form a cavity matching the canal space, and Mesh Modeling tools were used to refine mold dimensions for a tight, precise fit. A channel exactly parallel to the canal axis was voided within the mold to permit later insertion of a fiber post during setting of the bioceramic (Figure 5).

All digital procedures—baseline CBCT void-volume measurements, conversion of digital impressions into lock-and-key molds, and void segmentation in Mimics (Version 28.0)—were performed by two operators (M.K.A. and W.J.), each with five years of experience in digital dentistry.

**Technique D—Conventional (indirect) technique.** Following the previously reported case-report method, a light-body rubber impression of the canal was recorded as in Technique B but was not digitized. Instead, the light-body impression was embedded in a heavy-body putty rubber block of the same system (Zetaplus; Zhermack, Badia Polesine, Italy). After the putty had set, the soft-rubber impression was removed, and the resulting mold was filled directly with the bioceramic cement, which was allowed to set to form the inlay.

**Bio-Root Inlay fabrication.** The digitally designed lock-and-key molds (Techniques A–C) were 3D-printed with a masked stereolithography (MSLA) resin printer (ELEGOO Mars; ELEGOO Inc., Shenzhen, China) using a standard photopolymer model resin, then post-cured in the manufacturer’s UV wash-and-cure unit (ELEGOO Mercury, Shenzhen, China). All molds (A–D) were packed with a pre-mixed calcium silicate-based bioceramic cement (FKG Dentaire SA, La Chaux-de-Fonds, Switzerland). A glass-fiber post was inserted into the bioceramic putty before setting and removed, leaving the post space centered within the inlay to maintain a precise post space. After complete setting of the bioceramic, the fiber post was surface-treated and definitively luted within the inlay using a total-etch, dual-cure, hydrophobic resin cement, which has previously been shown to provide the highest bond strength between fiber posts and BioCeramic putty. All 40 inlays were fabricated by a single operator (Y.A.T.), a pediatric dentist with nine years of experience in endodontic treatment of immature permanent teeth in children, to standardize the fabrication procedure and eliminate inter-operator variability.

### 2.5. Void-Percentage Evaluation as an Outcome Measurement

**Figure 6:**
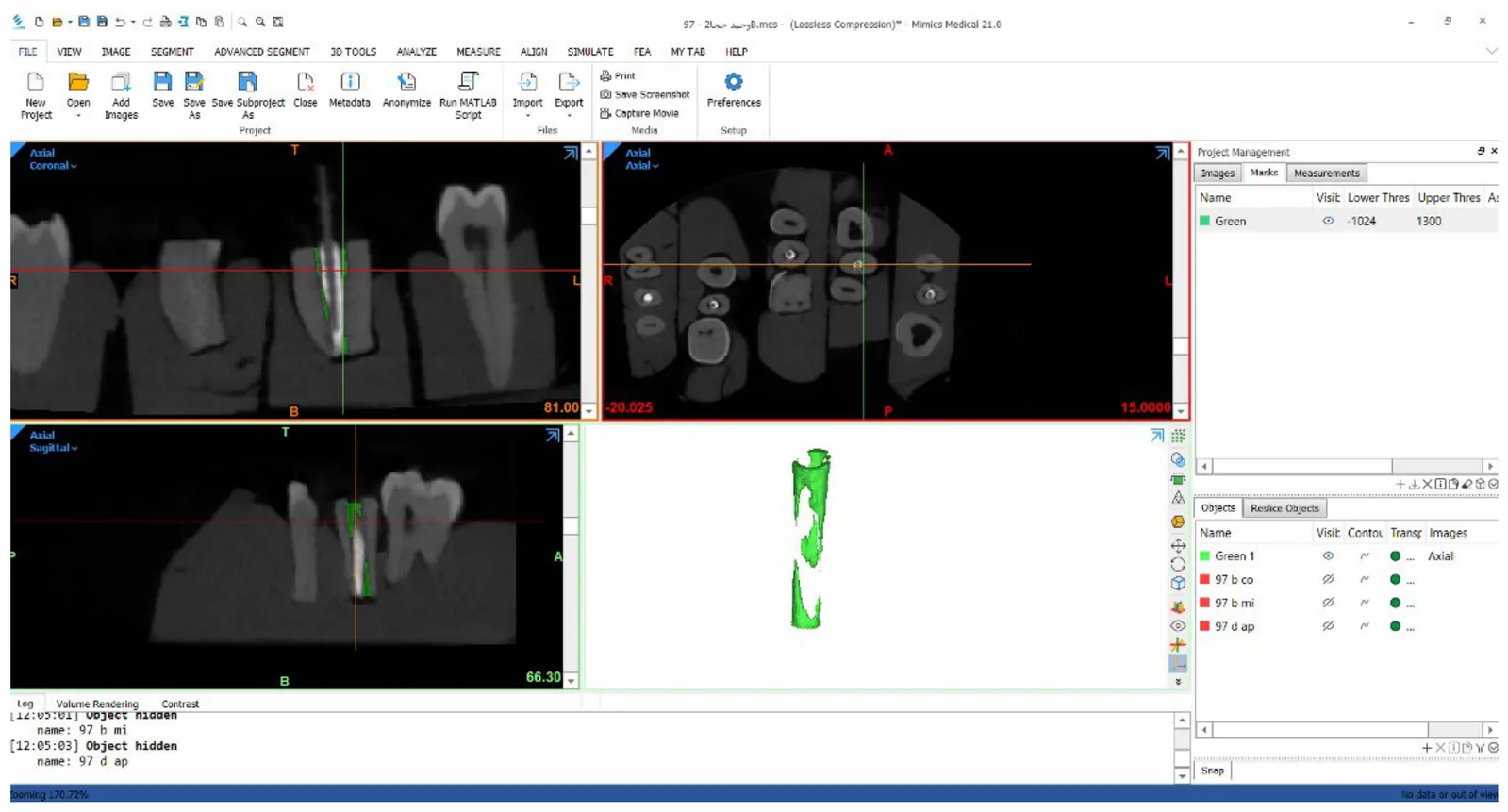
Isolation of the void volume in one specimen using Mimics software.

Baseline CBCT scans of all ten empty, prepared canals were acquired (PHT-6500; Vatech, Hwaseong, Republic of Korea), and the total canal volume was segmented in Mimics Research (v21.0.0.406) using the CBCT canal-impression procedure described in Technique C. After the inlays produced by each technique were seated, the roots were re-scanned using the same CBCT protocol, and the residual void volume around each inlay was segmented at the coronal, middle, and apical thirds (Figure 6). The void percentage was calculated as the residual void volume divided by the corresponding baseline (empty) canal volume.

The specimens were positioned on the CBCT plate in the same reproducible position for every scan at baseline (empty canals) and after seating each technique’s inlay, ensuring identical orientation across all scans of a given specimen for accurate volumetric comparison.

Void volumes in every CBCT section of each specimen after Bio-Root inlay insertion were measured jointly by two operators (M.T.A. and N.B.), each with ten years of experience in digital dentistry, who were blinded to the impression and Bio-Root inlay fabrication technique. To assess reliability, the measurements were repeated after a two-week interval, demonstrating excellent test–retest reliability (test–retest intraclass correlation coefficient, ICC = 0.92).

### 2.6. Statistical Analysis

Data were analyzed using SPSS (Version 20, IBM SPSS Inc., Chicago, IL, USA). Two complementary analytical strategies were adopted. The linear mixed model was pre-specified as the primary inferential analysis, as it was the only approach able to simultaneously model the repeated within-specimen dependency, the technique × canal-level interaction, and the canal-volume covariate, a dependency structure that non-parametric tests cannot accommodate. The model was fitted with technique, canal level, and their interaction as fixed effects, original canal volume as a covariate, and specimen (tooth) as a random intercept.

The validity of the mixed model was verified through diagnostics performed on the model residuals rather than on the raw observations, since it is the residuals, not the raw data, that are assumed to be normally distributed in a linear mixed model. The Shapiro–Wilk test on the residuals indicated no significant departure from normality (W = 0.981, *p* = 0.34); a normal Q–Q plot showed the residuals falling along the reference line, and a histogram confirmed an approximately symmetric distribution. Homoscedasticity was assessed by plotting the standardized residuals against the fitted values, which revealed a random scatter with no funnel-shaped pattern, supporting the assumption of constant variance.

The Friedman and Bonferroni-adjusted Wilcoxon signed-rank tests were pre-specified as secondary, confirmatory (sensitivity) analyses. Because the raw void-percentage values were bounded and non-normally distributed, as verified by the Shapiro–Wilk test applied within each technique × canal-level subgroup, these distribution-free methods tested whether the within-level differences among techniques held without reliance on any distributional assumption: the Friedman test was applied at each canal level, followed by Bonferroni-adjusted Wilcoxon signed-rank pairwise comparisons. Effect sizes were reported for these analyses: Kendall’s W for the Friedman tests and the matched-pairs rank-biserial correlation (r) for the Wilcoxon comparisons, with values of 0.1, 0.3, and 0.5 denoting small, moderate/medium, and large effects, respectively. All primary inferences are drawn from the mixed model, with the non-parametric results serving only to confirm its robustness; the convergence of the two approaches strengthens confidence in the findings. The significance level was set at α = 0.05.

## 3. Results

**Figure 7:**
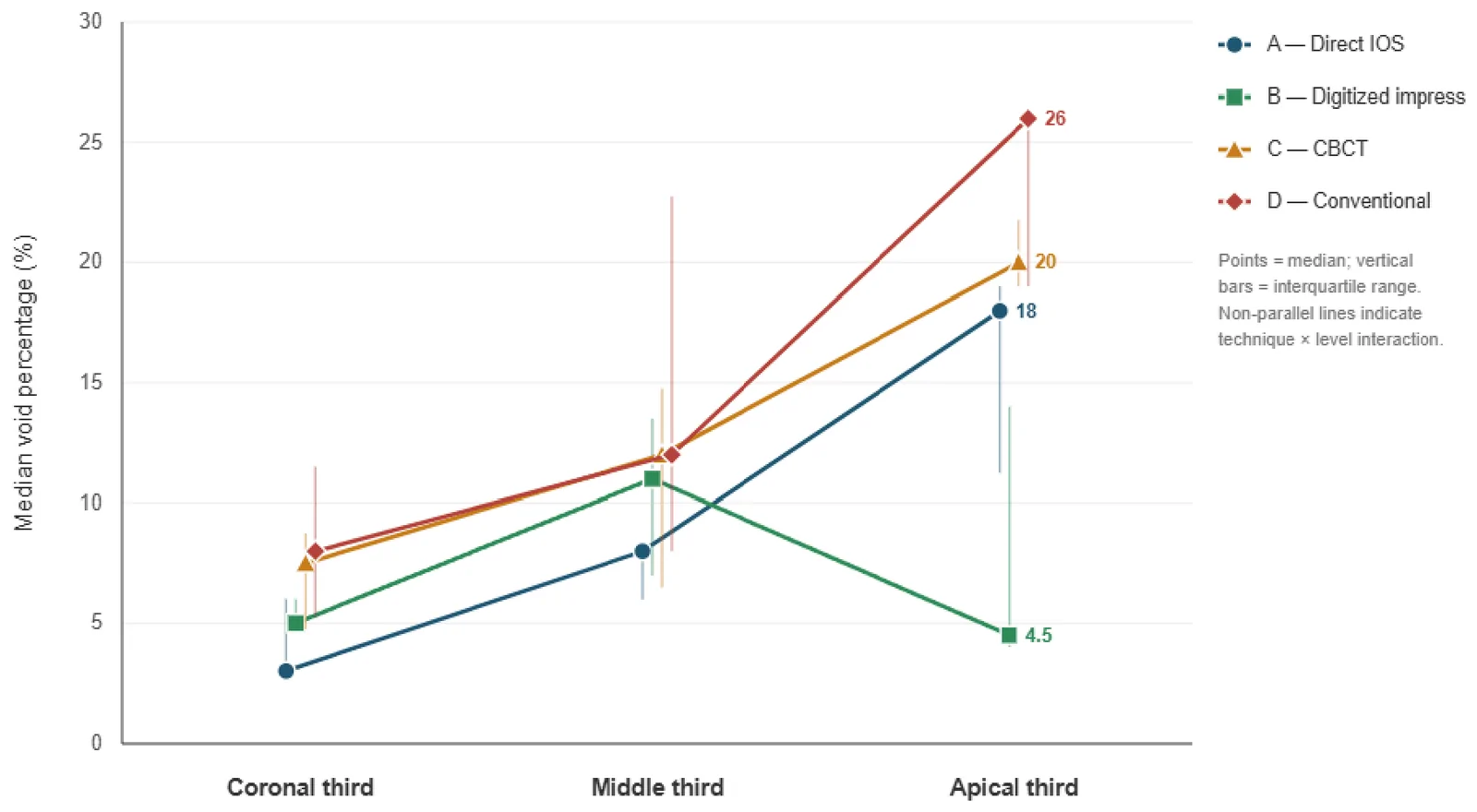
Technique × canal-level interaction plot (*n* = 10 specimens per technique at each canal level; 120 observations in total). Points represent the median void percentage for each technique at the coronal, middle, and apical thirds; vertical bars denote the interquartile range, and small horizontal offsets separate the four techniques at each level. The non-parallel and crossing trajectories illustrate the significant technique × canal-level interaction: the four techniques are closely grouped coronally but diverge toward the apex, where Technique B (digitized impression) improves markedly while Techniques C (CBCT) and D (conventional) deteriorate.

Overall, void percentage differed markedly among the four techniques. Technique B produced the lowest median void percentage, and Technique A the second lowest, whereas the CBCT-based (C) and conventional (D) techniques produced the largest voids. Void percentage also rose steadily from the coronal to the apical third, with the apex showing roughly twice the void of the coronal region (Table 1, Figure 7).

This apico-coronal pattern held across all techniques but varied in magnitude (Table 2). Coronally, all four techniques performed similarly well. In the middle third, voids increased across the board, with Technique A showing the lowest void percentage. The techniques diverged most at the apex: Technique B maintained superior apical adaptation, Technique A deteriorated markedly, and Techniques C and D showed the largest apical voids.

The Friedman test confirmed these observations (Table 2): differences among techniques were non-significant coronally (*p* = 0.437) but significant in the middle third (*p* = 0.014) and, most strongly, at the apex (*p* < 0.001), as well as overall (*p* = 0.001). Bonferroni-adjusted Wilcoxon signed-rank comparisons (α = 0.0083) localized these differences to the apical third: Technique B differed significantly from both Techniques C and D, and Technique A from Technique C (all *p* = 0.007, r = 0.62), whereas the two poorest-performing techniques did not differ (C vs. D, *p* = 1.000). The overall analysis mirrored this pattern, chiefly separating the digital techniques (A and B) from the CBCT-based Technique C, and Technique B from Technique D.

A linear mixed model was fitted with technique, canal level, and their interaction as fixed effects, canal volume as a covariate, and specimen (tooth) as a random intercept to account for repeated within-specimen measurements (Table 3). The technique × canal level interaction was significant (F(6,98) = 3.67, *p* = 0.002), indicating that the difference among techniques was not constant across canal levels. Because of this interaction, neither the technique nor the canal level main effect is interpreted in isolation; instead, the analysis focuses on the simple effects of technique within each canal level (Table 2). The interaction itself indicates only that the technique differences vary by region—it does not, on its own, identify which region is most affected. That determination rests on the within-level comparisons: technique differences were absent coronally (Friedman *p* = 0.437), emerged in the middle third (*p* = 0.014), and were both largest and statistically strongest at the apex (*p* < 0.001), where Technique B differed significantly from both Techniques C and D and Technique A from Technique C (all *p* = 0.007, r = 0.62). It is these apical simple-effect comparisons—not the interaction term—that identify the apex as the region where the choice of impression technique is decisive. For completeness, the canal level effect was also significant (F(2,98) = 5.82, *p* = 0.004), consistent with the apico-coronal increase in voids, but in the presence of the interaction this main effect is reported descriptively rather than interpreted independently. Canal volume had no independent effect once canal level was accounted for (F(1,98) = 0.01, *p* = 0.928). The random specimen intercept accounted for only a small share of the total variability (specimen variance = 1.28; residual = 27.35; variance-partition ICC = 0.045), and the ranking of the techniques remained consistent across specimens.

## 4. Discussion

Digital workflows have transformed multiple domains of dentistry, including guided implant placement, endodontic microsurgery, guided negotiation of calcified canals, and post-and-core fabrication [15,16]. Their principal advantages, including reduced treatment time, simplified procedures, and improved patient comfort, have been repeatedly documented [17,18]. The present study extended this concept to apexification by, for the first time, evaluating the three-dimensional adaptation of prefabricated Bio-Root inlays fabricated through four different impression pathways. The null hypothesis, which stated that all methods would yield comparable adaptation, was rejected.

A light-body rubber impression, subsequently digitized with an intraoral scanner, produced the lowest median void percentage, followed by the direct intraoral scan. By contrast, the CBCT-designed and fully conventional inlays showed the poorest adaptation. This gradient indicates that capturing canal geometry via an optical surface (whether directly or via a physical replica) reproduces the internal anatomy of the immature canal more faithfully than volumetric radiographic segmentation or purely analog molding.

Although micro-computed tomography (micro-CT) offers superior spatial resolution, CBCT was selected for this volumetric analysis for two reasons: (i) it directly replicates the clinical imaging modality most readily available to practitioners for postoperative assessment, thereby enhancing the translational relevance of the findings; and (ii) the standardized canal volume (mean 14.30 mm^3^) was sufficiently large relative to the 0.2 mm voxel size to permit reliable segmentation of clinically relevant void spaces.

Although CBCT was used both as the image source for Technique C and as the outcome assessment tool, the two applications were fundamentally different. CBCT served as the imaging modality during model fabrication, whereas adaptation was quantified by independent segmentation of the residual void volume after inlay placement, with assessment performed in a blinded manner. Nevertheless, the inherent voxel-size limitation of CBCT should be acknowledged as a potential source of measurement uncertainty.

A consistent and clinically important finding was the apico-coronal deterioration in adaptation across all techniques: the median void percentage rose from 6.00% coronally to 11.00% in the middle third and 19.00% apically, with the four techniques differing negligibly in the coronal third—where access and light penetration are unrestricted—but diverging in the middle and, most markedly, the apical third. This apex-dependent divergence was formally confirmed by the significant technique × canal level interaction in the mixed model (F(6,98) = 3.67, *p* = 0.002), establishing that the superiority of a given impression technique is conditional on canal region rather than uniform along the root. Clinically, this apical advantage is the most consequential outcome of the study, since the apex is the region the operator can neither see nor instrument directly, and the seal established there governs the long-term prognosis of an immature tooth: the digitized-impression and direct-scan techniques reproduced the widest, least accessible part of the canal closely enough to leave only a thin residual space, whereas the CBCT-based and conventional techniques left substantially larger apical voids. In practical terms, a tighter apical adaptation means less reliance on sealer to compensate for bulk discrepancies, a more predictable apical stop against which the bioceramic can set, and a reduced pathway for bacterial percolation all of which matter far more at the apex of an open-apex tooth than in the coronal third [19], where adaptation was uniformly good regardless of technique. The apex therefore represents the decisive region for technique selection, and the superior apical performance of the digital workflows translates directly into a clinically more favorable seal where it is needed most.

Beyond resolution limits, an important methodological caveat must be acknowledged: CBCT served dual roles, both as the source image for designing Technique C and as the imaging modality for assessing the outcome. Although these were fundamentally different processes—segmentation of the canal space for design versus segmentation of the residual void for assessment—and were conducted independently by blinded operators, the shared imaging platform could theoretically introduce a systematic bias. Specifically, inherent CBCT thresholding errors might affect both the fabricated inlay and the void measurement in the same direction. Future studies employing micro-CT as the gold-standard reference for outcome assessment are warranted to validate these findings.

The two digital techniques diverged sharply at the apex. Technique A, highly accurate coronally and in the middle third, lost precision apically, whereas Technique B retained excellent apical adaptation and was significantly better than both the CBCT and conventional techniques in this region. This divergence is best explained by the optical path of intraoral scanners: the scanner’s beam and field of view are progressively obstructed within a narrow, deep canal, so the apical wall is captured incompletely during direct scanning. In contrast, the light-body rubber first flows into and records the apical geometry as a physical replica, which is then digitized under unobstructed external conditions, effectively decoupling anatomical capture from the optical limitations of intracanal scanning. This interpretation aligns with previous evidence that the accuracy of direct intraoral scanning of post spaces is acceptable only up to a limited depth and decreases as the prepared length increases [8].

The comparatively poor performance of the CBCT-based technique may be attributable to the intrinsic resolution of volumetric imaging. Even at a 0.2 mm voxel size, partial-volume averaging and Hounsfield unit thresholding introduce boundary uncertainty at the fine, tapering apical walls, so the segmented cavity deviates from true canal morphology more than an optical surface scan. Although CBCT has proven valuable in improving endodontic practice, its voxel-limited spatial resolution appears insufficient for the sub-millimeter fit required of a prefabricated apical inlay. The conventional technique showed the greatest voids overall, reflecting the accumulation of dimensional error inherent in a fully analog, multi-step molding sequence performed without digital refinement of undercuts and internal irregularities. In particular, seating the light-body rubber impression into the heavy-body putty block may itself distort the soft impression, introducing dimensional inaccuracies that are subsequently transferred to the final inlay.

The small proportion of variance attributable to the random specimen effect (ICC = 0.045) indicates that void formation was governed chiefly by the impression technique and canal level rather than by between-tooth anatomical variability, strengthening the generalizability of the observed differences. Moreover, canal volume had no independent effect on void percentage once canal level was accounted for, indicating that the technique itself, rather than canal size, was the dominant driver of adaptation.

These findings should be interpreted in the context of the broader digital-versus-conventional debate. In full-arch implant impressions, conventional techniques have been reported to outperform digital ones [20]; yet in the confined, single-canal geometry examined here, digital surface capture proved superior. This contrast underscores that the accuracy of any impression modality is context-dependent, governed by the accessibility and scale of the anatomy recorded. For the immature canal specifically, a digitized physical impression offers a practical compromise that combines the apical fidelity of a flowable material with the design flexibility of a digital workflow.

Clinically, the results suggest that a digitized soft-rubber impression is currently the most reliable method for fabricating well-adapted prefabricated Bio-Root inlays, particularly when apical sealing is paramount. Direct intraoral scanning remains an attractive, chairside-efficient alternative for the coronal and middle thirds but may require adjunctive strategies to overcome its apical limitations.

From a clinical standpoint, a certain proportion of voids at the inlay–canal interface appears unavoidable regardless of the impression technique. In practice, the prefabricated Bio-Root inlay is seated with a bioceramic sealer from the same material family, which fills residual voids and ensures a continuous, homogeneous interface with comparable physicochemical and biological behavior. Nevertheless, the sealer film should always be kept as thin as possible: the inlay is designed to occupy the bulk of the canal space, while the sealer serves only to obturate the small, unavoidable gaps, since bond strength and dimensional stability are generally more favorable with a thin sealer layer than with a thick one.

To contextualize the present data: for an average canal volume of 14.30 mm^3^, an apical void of approximately 20–26% (the median observed in the poorest-performing groups, Techniques C and D) corresponds to roughly 2.9–3.7 mm^3^ of residual space. This volume is readily accommodated by a flowable bioceramic sealer; however, a sealer film of this thickness approaches the upper limit recommended for optimal dimensional stability and bond strength. Thus, while Technique B’s apical median void of 4.50% (approx. 0.6 mm^3^) offers a superior safety margin, the clinical significance of the absolute difference between techniques warrants further investigation through long-term leakage or push-out bond strength studies.

The main limitation of this study was that it was an in vitro study using standardized, artificially created immature canals in extracted mature premolars, which cannot fully replicate the irregular anatomy, moisture, and blood contamination of the clinical immature apex. Void percentage was assessed by CBCT, whose resolution imposes a measurement floor on the smallest detectable voids; higher-resolution micro-CT could refine future quantification. The crossover design controlled for inter-tooth variability but did not evaluate long-term sealing, push-out bond strength, or clinical outcomes.

Future research should validate these workflows on anatomically variable, immature natural teeth, incorporate micro-CT or fluid-filtration sealing assessments, and explore hybrid capture strategies, such as combining an apical rubber replica with a coronal direct scan, to maximize adaptation throughout the canal. Specifically, we propose that subsequent investigations use high-resolution micro-CT (voxel size ≤ 20 µm) to directly compare the volumetric accuracy of the digitized impression (Technique B) with that of the direct intraoral scan (Technique A), with a particular focus on quantifying the exact apical depth at which direct optical scanning loses fidelity.

## 5. Conclusions

Within the limitations of this controlled in vitro study, the impression method significantly affected the three-dimensional adaptation of prefabricated Bio-Root inlays in simulated immature roots. Digital surface-capture pathways outperformed both CBCT-based design and the conventional technique, and adaptation deteriorated progressively toward the apex. A light-body rubber impression digitized with an intraoral scanner provided the best overall and apical adaptation, suggesting it may be the most promising approach for a digital apexification workflow. Direct intraoral scanning offered excellent coronal and middle-third fit but reduced apical accuracy.

## Figures and Tables

**Table 1:** Descriptive statistics (median (IQR) and range) of void-percentage values across techniques and root canal thirds.

Variable	Group	Median	IQR	Range (%)
*Techniques*	A	8.00	8.75	1–25
	B	6.00	7.00	1–19
	C	12.00	12.50	4–31
	D	12.00	19.00	3–31
*Root canal thirds*	Coronal	6.00	4.50	1–15
	Middle	11.00	6.50	2–27
	Apical	19.00	10.75	2–31

Descriptive statistics only (N = 10 specimens). Each canal-level measurement is an individual observation (not summed or averaged per tooth); per-technique medians pool 30 observations (10 specimens × 3 levels) and per-level medians pool 40 observations (10 specimens × 4 techniques). Inferential comparisons are reported in Table 2 and Table 3: differences among techniques within each level in Table 2, and the canal-level effect.

**Table 2:** Void percentage (%) by technique at each root canal third and overall, expressed as median (IQR), with Friedman omnibus test and Bonferroni-adjusted Wilcoxon post hoc comparisons.

Level	Technique	Median	IQR	χ^2^	df	*p*-Value	Kendall’s W	Significant Post Hoc Comparisons (Wilcoxon, α = 0.0083)
Coronal	A	3.00 ^a^	3.00	1.29	3	0.437	0.043	—
	B	5.00 ^a^	1.00					
	C	7.50 ^a^	4.00					
	D	8.00 ^a^	6.25					
Middle	A	8.00 ^a^	2.00	10.26	3	**0.014**	0.342	A–C: *p* = 0.012, r = 0.55; A–D: *p* = 0.015, r = 0.56
	B	11.00 ^ab^	6.50					
	C	12.00 ^b^	8.25					
	D	12.00 ^b^	14.75					
Apical	A	18.00 ^a^	7.75	19.77	3	**<0.001**	0.659	A–B: *p* = 0.025, r = 0.49; A–C: *p* = 0.007, r = 0.62; A–D: *p* = 0.021, r = 0.53; B–C: *p* = 0.007, r = 0.62; B–D: *p* = 0.007, r = 0.62
	B	4.50 ^b^	10.00					
	C	20.00 ^c^	2.75					
	D	26.00 ^c^	7.00					
Overall	A	8.00 ^a^	8.75	19.23	3	**0.001**	0.641	A–C: *p* = 0.004, r = 0.47; A–D: *p* = 0.021, r = 0.45; B–C: *p* = 0.007, r = 0.40; B–D: *p* = 0.013, r = 0.50
	B	6.00 ^a^	7.00					
	C	12.00 ^b^	12.50					
	D	12.00 ^b^	19.00					

Data are median void percentage (%) with interquartile range (IQR). χ^2^, df and *p*-value are from the Friedman test (df = 3); Kendall’s W is the corresponding effect size (0.1, 0.3, 0.5 = small, moderate, large). Within each level, medians not sharing a common superscript letter differ significantly by Bonferroni-adjusted Wilcoxon signed-rank test (α = 0.0083); r is the matched-pairs rank-biserial correlation (effect size). Only statistically significant pairwise comparisons are listed; “—” denotes no significant pairs (or a non-significant omnibus test). Bold *p*-values indicate statistical significance.

**Table 3:** Linear mixed-model analysis of void percentage; fixed-effect estimates (%) with 95% CI, and variance components. Descriptive data elsewhere are reported as median (IQR), whereas the mixed model is estimated on the untransformed values.

***Fixed effects (references = Technique A, coronal third; model: void % ~ technique + canal level + technique × canal level + canal volume + (1|specimen))***					
**Term**	**Estimate (%)**	**SE**	**95% CI**	**t (df = 104)**	***p*** **-Value**
Intercept (Technique A, coronal)	8.41	5.89	−3.27, 20.10	1.43	0.156
Technique B–A	−1.63	1.35	−4.31, 1.04	−1.21	0.229
Technique C–A	4.40	1.35	1.72, 7.08	3.26	**0.002**
Technique D–A	5.50	1.35	2.82, 8.18	4.07	**<0.001**
Canal level: middle–coronal	4.52	2.52	−0.48, 9.52	1.79	0.076
Canal level: apical–coronal	10.07	3.29	3.56, 16.59	3.07	**0.003**
Canal volume (per mm^3^)	−0.03	0.30	−0.62, 0.57	−0.09	0.928
***Omnibus tests of the fixed effects (Type III)***					
**Effect**		**Num df**	**Den df**	**F**	***p*** **-Value**
Technique		3	98	12.87	**<0.001**
Canal level		2	98	5.82	**0.004**
Technique × Canal level		6	98	3.67	**0.002**
Canal volume		1	98	0.01	0.928
***Random effects (variance components)***					
**Component**		**Variance**	**SD**	**Proportion (ICC)**	
Specimen (random intercept)		1.28	1.13	0.045	
Residual		27.35	5.23		

Dependent variable: void percentage (%). Technique, canal level, and their interaction were modelled as fixed effects, original canal volume as a covariate, and specimen (tooth) as a random intercept. Because the technique × canal level interaction was significant (F(6,98) = 3.67, *p* = 0.002), the effect of technique on void percentage depended on the canal third; consequently the main effects are not interpreted in isolation, and techniques were compared within each canal level (Table 2). Canal level had a significant effect (F(2,98) = 5.82, *p* = 0.004), whereas canal volume had no independent effect after adjustment for canal level (F(1,98) = 0.01, *p* = 0.928). Fixed-effect denominator degrees of freedom were obtained by the containment method. The specimen random intercept accounted for only 4.5% of the total variance (ICC = 0.045). The fixed-effect coefficient estimates are reported from the additive model (technique + canal level + canal volume) for interpretability of the main-effect contrasts, and their *t*-tests carry 104 denominator degrees of freedom; the Type III omnibus F-tests and the technique × canal level interaction are derived from the full model including the interaction term (denominator df = 98).

## Data Availability

The original contributions presented in this study are included in the article. Further inquiries can be directed to the corresponding author.

## References

[1] Torabinejad M., Parirokh M. (2010). Mineral trioxide aggregate: A comprehensive literature review—Part II: Leakage and biocompatibility investigations. J. Endod..

[2] Alsayed Tolibah Y., Bshara N., Aljabban O., Abbara M.T., Alhaji M., Almasri I.A., Baghdadi Z.D. (2025). Randomized Trial of Bioceramic Apical Barrier Methods in Necrotic Immature Incisors: Effects on Pain, Extrusion, and Procedure Duration. Children.

[3] Prati C., Gandolfi M.G. (2015). Calcium silicate bioactive cements: Biological perspectives and clinical applications. Dent. Mater..

[4] Tolibah Y.A., Droubi L., Alkurdi S., Abbara M.T., Bshara N., Lazkani T., Kouchaji C., Ahmad I.A., Baghdadi Z.D. (2022). Evaluation of a Novel Tool for Apical Plug Formation during Apexification of Immature Teeth. Int. J. Environ. Res. Public Health.

[5] Rosaline H., Rajan M., Deivanayagam K., Reddy S.Y. (2018). BioRoot inlay: An innovative technique in teeth with wide open apex. Indian J. Dent. Res..

[6] Thiyagarajan G., Manoharan M., Veerabadhran M.M., Murugesan G., Vinodh S., Kamatchi M. (2023). Biodentine as BioRoot Inlay: A Case Report. Int. J. Clin. Pediatr. Dent..

[7] Alsayed Tolibah Y., Awad M.K., Najjar Y.M., Abbara M.T., Almonakel M.B., Abou Nassar J., Aljabban O., Bshara N. (2025). Effects of Different Cementation Systems on Pull-out Bond Strength of Fibre Post to Bioceramic Putty Using a 3D Prefabricated Root Canal Model of Immature Permanent Teeth: An In-Vitro Study. Eur. Endod. J..

[8] Almalki A., Conejo J., Kutkut N., Blatz M., Hai Q., Anadioti E. (2024). Evaluation of the accuracy of direct intraoral scanner impressions for digital post and core in various post lengths: An in-vitro study. J. Esthet. Restor. Dent..

[9] Dupagne L., Mawussi B., Tapie L., Lebon N. (2023). Comparison of the measurement error of optical impressions obtained with four intraoral and one extra-oral dental scanners of post and core preparations. Heliyon.

[10] Tchorz J.P., Gierl V., Piasecki L., Frank W., Wrbas K.T. (2023). Effects of CBCT acquisition protocol and additional superimposed computerized optical impressions on the accuracy of root canal length measurements: An ex vivo study. Int. J. Comput. Dent..

[11] Mohamed R.H., Abdelrahman A.M., Sharaf A.A. (2022). Evaluation of rotary file system (Kedo-S-Square) in root canal preparation of primary anterior teeth using cone beam computed tomography (CBCT)—In vitro study. BMC Oral Health.

[12] Fornara R., Pisano M., Salvati G., Malvicini G., Iandolo A., Gaeta C. (2024). Management of Calcified Canals with a New Type of Endodontic Static Guide: A Case Report. Dent. J..

[13] Pouhaër M., Picart G., Baya D., Michelutti P., Dautel A., Pérard M., Le Clerc J. (2022). Design of 3D-printed macro-models for undergraduates’ preclinical practice of endodontic access cavities. Eur. J. Dent. Educ..

[14] Juha W., Sarkis E., Alsayed Tolibah Y. (2024). Three-dimensional assessment of obturation volume in lateral canals after three obturation techniques with bioceramic sealer: An in vitro comparative study. BDJ Open.

[15] Nagendrababu V., Murray P.E., Ordinola-Zapata R., Peters O.A., Rôças I.N., Siqueira J.F., Priya E., Jayaraman J., Pulikkotil S.J., Camilleri J. (2021). PRILE 2021 guidelines for reporting laboratory studies in Endodontology: A consensus-based development. Int. Endod. J..

[16] Pandolfo M.T., Rover G., Bortoluzzi E.A., Teixeira C.D.S., Rossetto H.L., Fernades P.C.D.S.V., Côrte-Real I.S.G., Carvalho S.M.F., Garcia L.D.F.R. (2021). Fracture Resistance of Simulated Immature Teeth Reinforced with Different Mineral Aggregate-Based Materials. Braz. Dent. J..

[17] Joda T., Zarone F., Ferrari M. (2017). The complete digital workflow in fixed prosthodontics: A systematic review. BMC Oral Health.

[18] Mangano F., Gandolfi A., Luongo G., Logozzo S. (2017). Intraoral scanners in dentistry: A review of the current literature. BMC Oral Health.

[19] Bukhary S. (2025). Apexification of an Endodontically Failed Permanent Tooth with an Open Apex: A Case Report with Histologic Findings. Medicina.

[20] Jasim A.G., Abo Elezz M.G., Altonbary G.Y., Elsyad M.A. (2024). Accuracy of digital and conventional implant-level impression techniques for maxillary full-arch screw-retained prosthesis: A crossover randomized trial. Clin. Implant Dent. Relat. Res..

